# Compounds enhancing human sperm motility identified using a high-throughput phenotypic screening platform

**DOI:** 10.1093/humrep/deac007

**Published:** 2022-01-20

**Authors:** Franz S Gruber, Zoe C Johnston, Neil R Norcross, Irene Georgiou, Caroline Wilson, Kevin D Read, Ian H Gilbert, Jason R Swedlow, Sarah Martins da Silva, Christopher L R Barratt

**Affiliations:** 1 National Phenotypic Screening Centre, School of Life Sciences, University of Dundee, Dundee, UK; 2 Reproductive Medicine Research Group, Division of Systems Medicine, School of Medicine, Ninewells Hospital and Medical School, University of Dundee, Dundee, UK; 3 Drug Discovery Unit, Division of Biological Chemistry and Drug Discover, Wellcome Centre for Anti-Infectives Research, University of Dundee, Dundee, UK; 4 Division of Computational Biology and Centre for Gene Regulation and Expression, School of Life Sciences, University of Dundee, Dundee, UK

**Keywords:** drug discovery, sperm motility, high-throughput screening, spermatozoa, subfertility

## Abstract

**STUDY QUESTION:**

Can a high-throughput screening (HTS) platform facilitate male fertility drug discovery?

**SUMMARY ANSWER:**

An HTS platform identified a large number of compounds that enhanced sperm motility.

**WHAT IS KNOWN ALREADY:**

Several efforts to find small molecules modulating sperm function have been performed but none have used high-throughput technology.

**STUDY DESIGN, SIZE, DURATION:**

Healthy donor semen samples were used and samples were pooled (3–5 donors per pool). Primary screening was performed singly; dose–response screening was performed in duplicate (using independent donor pools).

**PARTICIPANTS/MATERIALS, SETTING, METHODS:**

Spermatozoa isolated from healthy donors were prepared by density gradient centrifugation and incubated in 384-well plates with compounds (6.25 μM) to identify those compounds with enhancing effects on motility. Approximately 17 000 compounds from the libraries, ReFRAME, Prestwick, Tocris, LOPAC, CLOUD and MMV Pathogen Box, were screened. Dose–response experiments of screening hits were performed to confirm the enhancing effect on sperm motility. Experiments were performed in a university setting.

**MAIN RESULTS AND THE ROLE OF CHANCE:**

From our primary single concentration screening, 105 compounds elicited an enhancing effect on sperm motility compared to dimethylsulphoxide-treated wells. Confirmed enhancing compounds were grouped based on their annotated targets/target classes. A major target class, phosphodiesterase inhibitors, were identified, in particular PDE10A inhibitors as well as number of compounds not previously known to enhance human sperm motility, such as those related to GABA signalling.

**LARGE SCALE DATA:**

N/A.

**LIMITATIONS, REASONS FOR CAUTION:**

Although this approach provides data about the activity of the compound, it is only a starting point. For example, further substantive experiments are necessary to provide a more comprehensive picture of each compound’s activity, the effect on the kinetics of the cell populations and subpopulations, and their potential mechanisms of action. Compounds have been tested with prepared donor spermatozoa, incubated under non-capacitating conditions, and only incubated with compounds for a relatively short period of time. Therefore, the effect of compounds under different conditions, for example in whole semen, for longer incubation times, or using samples from patient groups, may be different and require further study. All experiments were performed *in vitro*.

**WIDER IMPLICATIONS OF THE FINDINGS:**

This phenotypic screening assay identified a large number of compounds that increased sperm motility. In addition to furthering our understanding of human sperm function, for example identifying new avenues for discovery, we highlight potential compounds as promising start-point for a medicinal chemistry programme for potential enhancement of male fertility. Moreover, with disclosure of the results of screening, we present a substantial resource to inform further work in the field.

**STUDY FUNDING/COMPETING INTEREST(S):**

This study was supported by the Bill and Melinda Gates Foundation, Scottish Funding Council and Scottish Universities Life Science Alliance. C.L.R.B. is Editor for RBMO. C.L.R.B. receives funding from Chief Scientists Office (Scotland), ESHRE and Genus PLC, consulting fees from Exscientia and lecture fees from Cooper Surgical and Ferring. S.M.d.S. is an Associate Editor of *Human Reproduction*, and an Associate Editor of *Reproduction and Fertility*. S.M.d.S. receives funding from Cooper Surgical and British Dietetic Society. No other authors declared a COI.

## Introduction

Sperm dysfunction is the single most common cause of infertility. However, there is an absence of new diagnostic tools and non-medically assisted reproduction (MAR) based treatments for the subfertile man (Barratt *et al.*, 2017; De Jonge and Barratt, 2019). A fundamental obstacle is the relative paucity of knowledge of the production, formation, maturation and physiological functions of both the normal and dysfunctional spermatozoon. There is an urgent need to address this knowledge gap to formulate appropriate diagnostic assays, develop rational therapies and understand how external factors, such as the environment, influence these processes (Barratt *et al.*, 2021).

Although some progress has been made in our understanding of the workings of the mature spermatozoon using tools such as proteomics, electrophysiology and imaging, one area in which there has been minimal progress is the development of an effective high-throughput screening (HTS) system using motile human spermatozoa (Martins da Silva *et al.*, 2017). Current methods for assessment of sperm quality are time-consuming and inappropriate for high-throughput drug discovery (Schiffer *et al.*, 2014; Tardif *et al.*, 2014). One way around this has been to utilize HTS assays with surrogate measures of sperm function such as intracellular calcium concentration [Ca^2+^]_i_ (Schiffer *et al.*, 2014; Martins da Silva *et al.*, 2017). Although informative, these do not directly assess cell function and have key limitations. For example, [Ca^2+^]_i_ should not be used as a surrogate of sperm motility, as a number of compounds can generate an increase in [Ca^2+^]_i_ but have no significant effect on motility (Martins da Silva *et al.*, 2017; McBrinn *et al.*, 2019). To provide a transformative leap in understanding an HTS system for the assessment of quantitative motility is necessary. Recently, a phenotypic platform has been developed which examined human sperm motility in a high-throughput manner (Gruber *et al.*, 2020; Johnston *et al*., 2022).

Although this HTS system has been used to identify potential compounds for male contraception, i.e. those having a negative effect on human sperm function, it can conversely be used to uncover elements of sperm cell biology and function, and to identify compounds that enhance sperm function. For example, it allows for large scale screening of not only approved drugs, target-class specific libraries (such as ion channels, kinase inhibitors) but also large libraries of chemically diverse lead-like small molecules that could provide the starting point for medicinal chemistry. In this study, we utilized this HTS system to examine six libraries incorporating ∼17 000 compounds with the dual aim of furthering our understanding of human sperm function and, generating possible starting points for a medicinal chemistry programme for potential enhancement of male fertility.

## Materials and methods

### Experimental design

**Figure 1.**
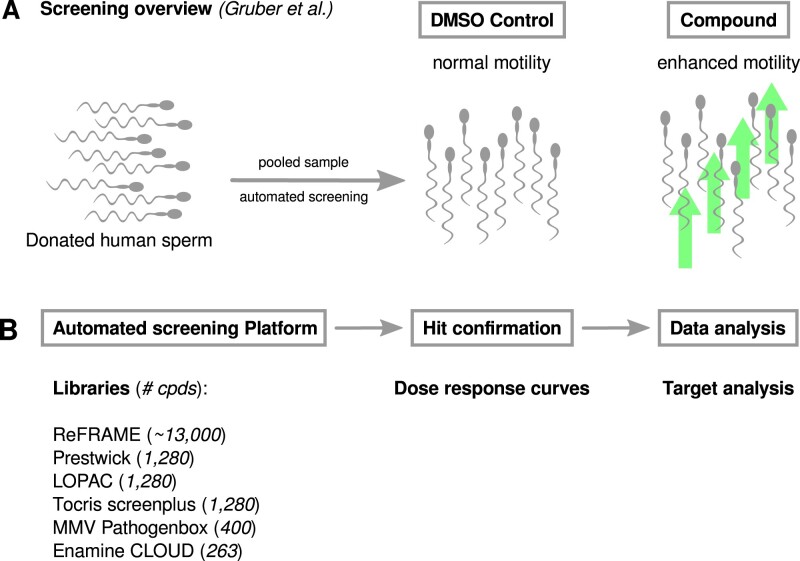
**Summary of screening platform and compound screening cascade.** (**A**) Motility screening overview as in Gruber *et al.* (2020). Donated human sperm are pooled and used for automated compound screening to detect compounds which increase sperm motility. DMSO is the vehicle control and the compound label represents a compound which increases motility (reflected by the green arrows). (**B**) Overview of screened compound libraries and follow-up steps. If a compound is selected as a potential hit in the initial screen, dose–response experiments are performed (hit confirmation). Analysis of the compounds with confirmed effects by a dose–response experiment provided some indication of potential target class (data analysis). DMSO, dimethylsulphoxide.

We used an HTS screening platform to assess the motility of live human spermatozoa. The platform and its development are described in detail in Gruber *et al.* (2020) and summarized below in brief. The platform was used to screen six compound libraries for their enhancing effects on motility. Whilst we have developed a screening module for detection of the acrosome reaction using flow cytometry (Gruber *et al.*, 2020), this was not examined in this study in order to increase throughput and focus on compounds affecting motility. The HTS system and experimental design are illustrated in Fig. 1.

### Selection and preparation of spermatozoa

Semen samples were obtained from volunteer donors. Written consent was obtained from each donor in accordance with the Human Fertilization and Embryology Authority (HFEA) Code of Practice (version 8) under local ethical approval (13/ES/0091) from the Tayside Committee of Medical Research Ethics B.

Donors had no known fertility problems and normal sperm concentrations, motility and semen characteristics according to Wolrd Health Organisation (WHO) criteria (2010). Samples were obtained by masturbation, after sexual abstinence of 2–5 days, and delivered to the research laboratory within 1 h of production. Samples were allowed to liquify at 37°C for 15–30 min prior to preparation by discontinuous density gradient centrifugation (DGC). Gradients were prepared using 80% and 40% Percoll (Sigma Aldrich, UK) diluted with non-capacitation media (Minimal Essential Medium Eagle Sigma M3024), supplemented with HEPES, sodium lactate and sodium pyruvate to achieve concentrations previously described (Tardif *et al.*, 2014).

For initial screening, prepared donor spermatozoa (80% fraction) were routinely pooled to create screening batches of three to five donors to reduce donor-to-donor variability. After preparation by DGC and pooling into screening batches, cells were incubated for 3 h at 37°C under non-capacitating conditions, as described previously (Gruber *et al.*, 2020).

### The high-throughput screening system

Full details of the HTS system, and its development, are discussed by Gruber *et al.* (2020). In brief, screening batches of cells were transferred to a robotic platform (HighRes Biosolutions Inc.) and maintained during the screen at 37°C. Assay-ready 384-well plates, containing compounds were prepared prior to the screen, and filled with ∼10 000 spermatozoa (20 μl) per well using a liquid handling system (MultiDrop Combi; ThermoFisher). These plates were incubated for 10 min prior to imaging. The HTS system utilized a Yokogawa CV7000 Cell Voyager high-throughput microscope to record time-lapse images from two positions in each well. An adaptation of a tracking algorithm, Trackpy v0.4.1 (Allan *et al.*, 2018), was utilized to track individual spermatozoa within each well and obtain kinematic parameters. Within the compound-test plates, dimethylsulphoxide (DMSO) was used as the vehicle control. Compounds in the plates were allocated at random.

### Libraries screened

The Pathogen box (generously provided by Medicines for Malaria Venture (MMV), https://www.mmv.org/mmv-open/pathogen-box/about-pathogen-box) is a small repurposing library assembled to screen against rare and neglected tropical diseases containing ∼400 diverse, drug-like molecules with demonstrated biological activity against different pathogens.

The CeMM Library of Unique Drugs (CLOUD) purchased from Enamine (https://enamine.net/hit-finding/compound-collections/bioreference-compounds/the-comprehensive-drug-collection-cloud) is a set of 263 small molecules representing the target and chemical space of FDA-approved drugs that has been used for drug repurposing.

Tocris compound library (Tocris, Bristol, UK, https://www.tocris.com/products/tocriscreen-plus_5840) comprises 1280 biologically active small molecule compounds.

LOPAC^®^1280LOPAC (Library of Pharmacologically Active Compounds https://www.sigmaaldrich.com/life-science/cell-biology/bioactive-small-molecules/lopac1280-navigator.html) composes a biologically annotated collection of inhibitors, receptor ligands, pharma-developed tools and approved drugs which impact many signalling pathways and covers all major drug target classes (1280 compounds).

Prestwick Chemical Library (http://www.prestwickchemical.com/libraries-screening-lib-pcl.html) comprising 1280 off patent drugs with high chemical and pharmacological diversity as well as known bioavailability and safety in humans (1280 compounds).

ReFRAME (Repurposing, Focused Rescue, and Accelerated Medchem) consists of ∼12 000 approved drugs, in-development small molecules and bio-active compounds in the initial library and was constructed as a library for potential drug repurposing (Janes *et al.*, 2018). The advantages of using this library was the potential to identify chemical compounds that are already approved for other indications or have undergone (or currently undergoing) clinical trials or have IND (Investigational New Drug) approval, hence potentially accelerating the progress towards a safe and effective enhancer of motility. CALIBR kindly provided up to 1% of hits for subsequent dose–response experiments. Only the structures of confirmed hits were unblinded. We also received a further 950 compounds, which were been added at a later stage to the ReFRAME collection (termed Reframe supplement).

### Data normalization and hit confirmation

All steps were performed as previously described (Gruber *et al.*, 2020). In summary, data from every compound well were normalized to those from in-plate DMSO controls (wells containing the same amount of DMSO as compound wells). Two positions were recorded in every well and the average of those positions was used for calculating % of control = (VCL median/DMSO median) × 100, where curvelinear velocity (VCL) median is the median of all sperm tracks (immotile, non-progressively motile, progressively motile) in each test well and DMSO median is the median of all 16 DMSO control wells on the corresponding plate. Hits from the primary screening were chosen based on percentage change compared to the control: 40% (for ReFRAME first batch, MMV Pathogenbox) or 20% (for LOPAC, CLOUD, Tocris, Prestwick, ReFRAME Supplement).

Hit compounds were examined in subsequent dose–response experiments. These consisted of two independent experiments utilizing different biological material (i.e. pooled spermatozoa samples from at least three different donors in each experiment). A compound was only identified as having a positive effect if it was confirmed in these dose–response experiments. Dose–response curves consisting of a series of 8-points with 3-fold dilution (with 10 µM top concentration) were fitted using the DR4PL (four-parameter logistic fit) package in R from which ECx (half-maximum effective concentration of × % effect) was estimated.

### Hit analysis

Chemical space was visualized by generating Morgan fingerprints using RDKit (radius = 2, bits = 2048) and using UMAP (Unifold Manifold Approximation and Projection) (McInnes *et al.*, 2018) for dimensionality reduction. Physico-chemical properties were calculated using RDKit (https://www.rdkit.org) and KNIME (Berthold *et al.*, 2008).

## Results

**Figure 2.**
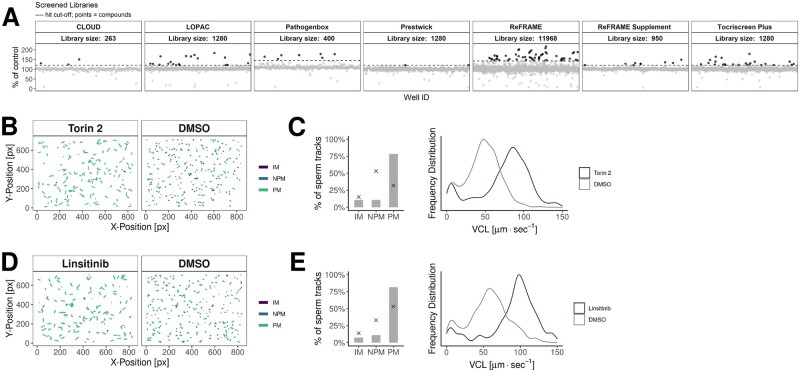
**Primary screening results with examples.** (**A**) Primary screening data from all screened libraries. Data are presented as a percentage of the control, which is defined as a well median VCL (including all sperm tracks) relative to a median VCL of vehicle (DMSO) control wells. Dashed lines represent hit cut-offs for each library. Each dot represents an individual compound and black dots represent hits with motility increase above the cut-off. (**B**) Example tracks of spermatozoa treated with Torin 2 or DMSO. The colour indicates the track class: IM (immotile), NPM (non-progressively motile) or PM (progressively motile). (**C**) Fraction of classified sperm tracks and frequency distribution of sperm track VCL of a well-treated with Torin 2. The *x* denotes a median of 16 DMSO wells in the same 384-well plate. (**D**) Example tracks of spermatozoa treated with Linsitinib or DMSO. (**E**) Fraction of classified sperm tracks and frequency distribution of sperm track VCL of a well-treated with Linsitinib. The *x* denotes a median of 16 DMSO wells in the same 384-well plate. DMSO, dimethylsulphoxide; VCL, curvelinear velocity.

**Figure 3.**
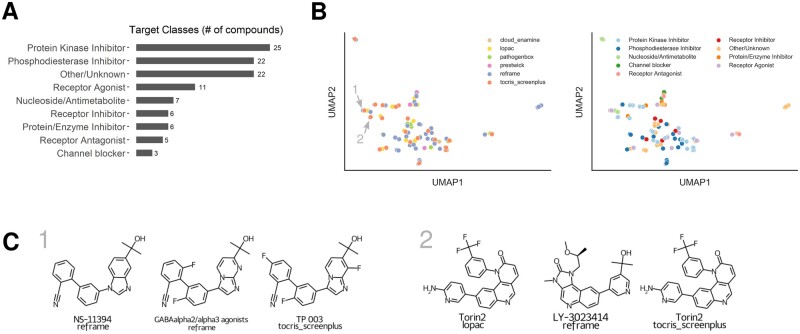
**Classification of screening results.** (**A**) Summary of the target classes of compounds confirmed by dose–response experiments. Target classes were identified according to library annotations. The ‘other/unknown’ category is comprised of compounds with no annotation available from the library vendor or unknown mode of action. (**B**) Chemical space visualization of motility enhancing compounds. Each enhancing compound has been encoded as chemical fingerprint (Morgan Fingerprint) with 2048 bit features. All features have been reduced to two dimensions using UMAP. The colour indicates screening library (left panel) or annotated target class (right panel). (**C**) Examples of similar hit structures with names and library information related to GABA signalling (Panel 1, also highlighted in B) and mTOR signalling (Panel 2, also highlighted in B). UMAP, Unifold Manifold Approximation and Projection.

In order to find compounds that positively enhance sperm motility, we used an HTS platform (Fig. 1A), to screen ∼17 000 compounds comprising a variety of small molecule libraries (Fig. 1B). Primary hits were identified based on percentage effect relative to DMSO control. These limits varied among the libraries and produced a hit rate between 0.3% and 1.9% (Table I). Primary hit compounds were confirmed in subsequent dose–response experiments and, in total 105 compounds were identified as confirmed hits (Supplementary Table SI), with moderate to high motility enhancing activity (Table I, Fig. 2A, Supplementary Table SI). Motility enhancing compounds shift the VCL of the population towards faster moving sperm cells compared to DMSO-treated wells (Fig. 2B–E, Supplementary Movie S1). The confirmed hits were annotated (broad definitions based on vendor annotations) to affect a variety of protein target classes (Fig. 3A, Supplementary Table SI).

**Table I:** Summary table of screened libraries.

Library	No. of compounds	No. of increaser hits	Hit cut-off^1^	% Hit rate
ReFRAME	∼12 000	37	∼40%^2^	0.3
ReFRAME supplement	∼950	9	20%	0.9
Prestwick chemical library	1280	4	20%	0.3
Tocriscreen plus	1280	24	20%	1.9
LOPAC	1280	20	20%	1.6
MMV pathogenbox	400	8	40%	2
CLOUD	263	3	20%	1.1
**Total**	**17 503**	**105**	**–**	**1.2**

1 Increase relative to DMSO control.

2 Max. 1% resupply.

DMSO, dimethylsulphoxide; MMV, Medicines for Malaria Venture; ReFRAME, Repurposing, Focused Rescue, and Accelerated Medchem.

Some compounds could not be assigned to a clear target class, however, of the annotated compounds, protein kinase inhibitors and phosphodiesterase inhibitors were the most common target classes (Fig. 3A). Another prominent target class were receptor modulators (inhibitor, agonist, antagonist), some of which are related to GABA signalling (Fig. 3A and C, Panel 1, Supplementary Table SI).

The most potent hits had sub-micromolar potency and substantial effects on motility (up to 190% compared to DMSO controls (Table II, Supplementary Table SI)). Chemical space visualization (Fig. 3B) reveals that several confirmed hits have similar or identical structures (Fig. 3C). The small molecule libraries used had some overlapping compounds and a number of these were either consistently active (e.g. SCH 58261, Torin2 (Fig. 3C, Panel 2) or Ethaverine) or consistently inactive (e.g. Rolipram, Milrinone or Sildenafil). A small number of compounds, for example Papaverine, were active in one library but not others.

**Table II:** Summary table of most potent screening compounds.

Compound	EC_X_ [µM]^1^	[% of control]	Target action
TAK-063	0.04	145	Phosphodiesterase inhibitor
RFM-012-216-7	0.14	128	Unknown
GW 843682X	0.17	192	Protein kinase inhibitor
Torin2	0.22	196	Protein inhibitor
Linsitinib	0.24	191	Receptor inhibitor
Tolafentrine	0.26	153	Phosphodiesterase inhibitor
Epetirimod	0.32	166	Unknown
E6005	0.33	151	Phosphodiesterase inhibitor
GABAalpha2/alpha3 agonists	0.35	138	Receptor agonist
JNJ-42396302	0.39	158	Phosphodiesterase inhibitor
NM-702	0.48	152	Phosphodiesterase inhibitor
RG-7203	0.49	162	Phosphodiesterase inhibitor
Trequinsin hydrochloride	0.5	143	Phosphodiesterase inhibitor
Dextofisopam	0.54	143	Unknown
Papverine	0.55	175	Phosphodiesterase inhibitor
LY-3023414	0.56	140	Protein kinase inhibitor
KF 15832	0.61	143	Phosphodiesterase inhibitor
STL515575	0.62	140	Unknown
Carbazeran	0.63	146	Phosphodiesterase inhibitor

1 Half maximal effective concentration.

**Figure 4.**
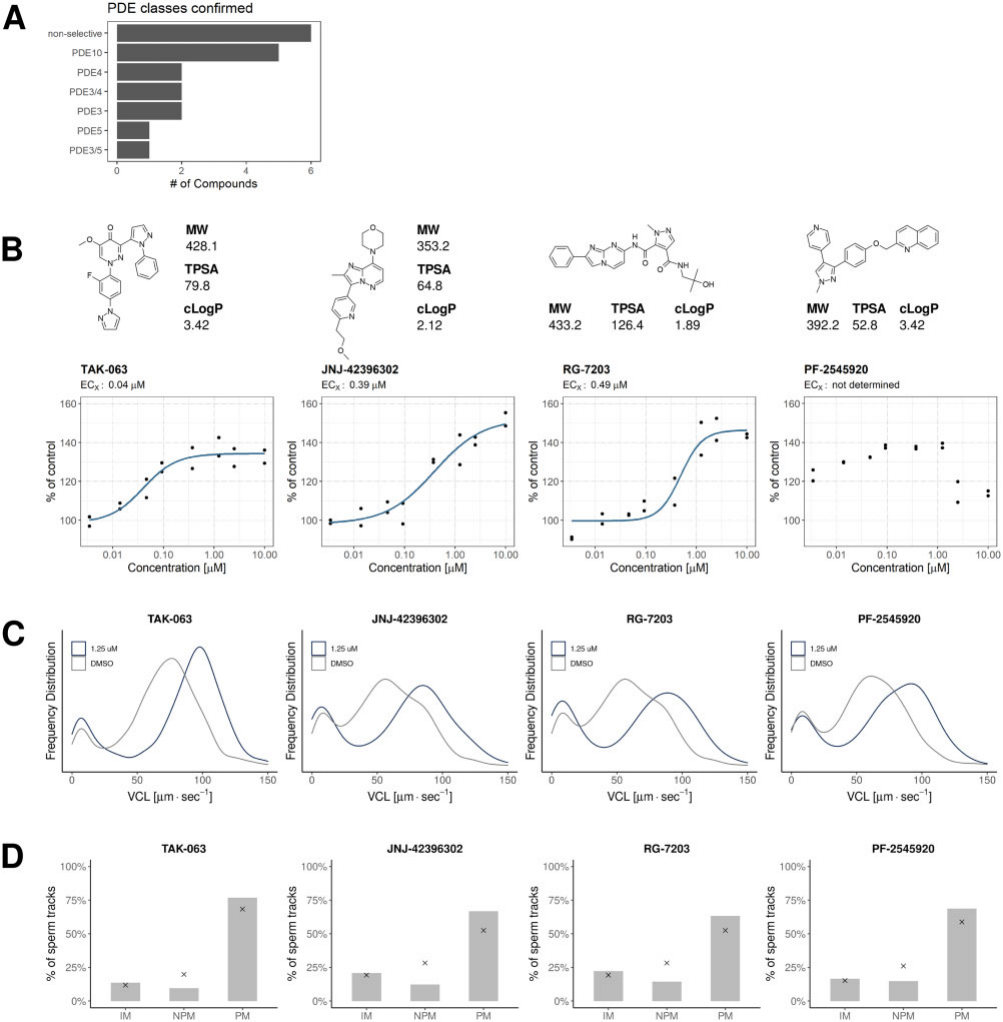
**Confirmation of PDE10A inhibitor hits.** (**A**) Summary graph of PDE inhibitor classes based on vendor annotation or available information resources (ChEMBL, PubChem, DrugBank). (**B**) Dose–response curves of four PDE10A inhibitors, with structures and physico-chemical properties. Blue line: four parameter logistic model. ECx, estimated half-maximum concentration. Each dot represents an individual data point, n = 2 for each concentration with data collected from two independent dose–response experiments utilizing different biological material (i.e. pooled spermatozoa samples from different donors in each experiment). Physico-chemical properties are defined as: MW, TPSA and cLogP. Note that no curve/ECx for PF-2545920 is shown due to noisy data at the two highest concentrations. (**C**) Frequency distributions of sperm VCL of each PDE10A inhibitor shown in (B) at 1.25 µM concentration (blue) compared to DMSO control wells (grey). (**D**) Fraction of classified sperm tracks for each PDE10A inhibitor at 1.25 µM concentration. The *x* denotes a median of 16 DMSO wells run in the same 384-well plate. cLogP, computed Crippen-LogP; DMSO, dimethylsulphoxide; MW, molecular weight; PDE, phosphodiesterase; TPSA, topological polar surface area; VCL, curvelinear velocity.

One prominent target class of confirmed hits were phosphodiesterase (PDE) inhibitors (Table II, Figs 3A and 4A). A number of these, TAK-063, JNJ-42396302, RG-7203 and PF-2545920 have an annotation of PDE10A inhibition (Table III, Fig. 4B) and showed sub-micromolar responses (0.04–0.49 µM) in dose–response experiments (Fig. 4B). All annotated PDE10A inhibitors used in this study had an enhancing effect on motility. For all four PDE10A inhibitors, a similar shift of VCL relative to DMSO controls was observed (Fig. 4C) and we observed an increase in the fraction of progressively motile cells (Fig. 4D). We did not detect any enhancing effect of compounds belonging to the methylxanthine class, e.g. IBMX or Pentoxifylline. Annotated PDE inhibitors used in this study are summarized in Supplementary Table SII.

**Table III:** Summary table of confirmed phosphodiesterase (PDE) inhibitors.

Compound	EC_X_ [µM]^1^)	[% of control]
TAK-063	0.04	145
JNJ-42396302	0.39	158
RG-7203	0.49	162
PF-2545920	n.d.^2^	130

1 Half maximal effective concentration.

2 EC_X_ not determined.

## Discussion

The current study utilized a validated imaging-based screening platform that measures fundamental aspects of human sperm behaviour (Gruber *et al.*, 2020) to screen a collection of chemical libraries comprising ∼17 000 approved drugs, clinically tested compounds and annotated chemical tool compounds for their potential to enhance motility. The aim was to further our understanding of human sperm function and generate possible start points for a medicinal chemistry programme for potential enhancement of male fertility.

There are significant challenges in producing a suitable platform for HTS of mature human spermatozoa (see Gruber *et al.*, 2020), and development is always a balance between achieving the necessary high throughput, assay robustness and a detailed assessment of each compound (see Johnston *et al.*, 2022). In these experiments, initial screening was performed under non-capacitating conditions at one concentration (6.25 µM) with compounds being assessed after a relatively short incubation (10–27 min). The data will therefore primarily reflect the use of these conditions and it is possible that other permutations, for example, screening under capacitating conditions, higher concentrations and/or longer incubation times may generate different results. In the current study, when a primary hit was identified, dose–response experiments were undertaken to confirm the hit and provide initial information on activity. Although this approach provides data about the activity of the compound, it is only a starting point. For example, further experiments are necessary to provide a more comprehensive picture of each compound’s activity and the effect on the kinetics of the cell populations and subpopulations and to determine their mechanisms of action (see McBrinn *et al.*, 2019 for examples of such investigations on human spermatozoa).

This study used spermatozoa from selected healthy donors rather than samples from patients. This was because, firstly, the aims of the study were to improve our understanding of the spermatozoon in addition to identifying possible compounds for further investigation; secondly, screening a large number of compounds requires substantial numbers of semen samples and, thirdly, donor samples are relatively homogenous. Future studies, using compounds, could subsequently utilize selected patient groups, e.g. those with oligozoospermia, isolated asthenozoospermia or oligoasthenozoospermia. These patient groups are heterogeneous with a variety of underlying causes (environment, genetic) and, as a consequence, different responses to the same or similar compounds are possible (see Tardif *et al.*, 2014).

The screening platform is complementary to a reductionist approach. Identification of several PDE inhibitors as confirmed hits in dose–response experiments (discussed below) provide evidence of the robustness of the HTS platform. Several phosphodiesterase inhibitors (PDEis) (e.g. ibudilast, trequinsin hydrochloride and papaverine) have previously been shown to significantly increase human sperm motility, confirming the ability of the HTS platform to identify compounds which are effective at or below concentrations of 6.25 μM. Furthermore, identical or related compounds which were present in two or more libraries were identified. For example, ibudilast was detected as a hit in both the LOPAC and Tocris libraries, and Trequinsin hydrochloride was confirmed in the ReFRAME and Tocris libraries. Another example is Torin2, a small molecule mTOR inhibitor, which was also detected in two libraries (LOPAC and Tocris) along with a structurally related compound (LY-3023414) with annotated activity against mTOR (see Figs 2B and C and 3C, Panel 2). This is intriguing as it has been recently described that in older men, mTORC1 is inhibited in highly motile spermatozoa compared to their defective/immotile counterparts (Silva *et al.*, 2019). For the largest library screened, the ReFRAME set, only 1% of the hits were unblinded, limiting our ability to analyse less active and inactive compounds from this set.

Of the target classes identified, PDE inhibitors (PDEi) account for 18/105 of the compounds found to increase sperm motility. This is not surprising and several of the PDEi hits have been previously identified to increase human sperm motility, e.g. Dipyrimadole, Ibudilast, Trequinsin Hydrochloride and Papaverine (Tardif *et al.*, 2014). Strikingly, a proportion of the PDEi hit compounds are annotated as specific to PDE10A. Although relatively little has been published on the effects of PDE10A inhibitors on human sperm, active PDE10A has been identified (Marechal *et al.*, 2017). Marechal confirmed their findings in additional experiments with the newly available PDE10A inhibitor MP-10 (Marechal *et al.*, 2017). MP-10, also known as Mardepodect or PF-2545920, was a hit in our screen (Supplementary Table SII). Little information is available for the other PDE10A inhibitors but the high representation of PDE10A inhibitors, combined with their apparent potency, could indicate their potential for further investigations for use in fertility treatment and or MAR.

Several PDE inhibitors which have been well documented for their effects on motility parameters of human sperm, including pentoxifylline aminophylline, theophylline, pentoxifylline, caffeine, and 3-Isobutyl-1-methylxanthine (IBMX) did not appear as hits (Supplementary Tables SI and SII). While this might initially be surprising, it is worth noting that initial screening conditions were at 6.25 μM for 10–27 min incubation and the actions of these drugs may require higher doses and/or longer incubation times. IBMX, for example, is used at concentrations from 30 μM to 1 mM (Lefievre *et al.*, 2000; Pons-Rejraji *et al.*, 2011; Tardif *et al.*, 2014; Marechal *et al.*, 2017). Similarly, pentoxifylline has been used at 3–4 mM (Tesarik *et al.*, 1992; Burger *et al.*, 2000; Patrizio *et al.*, 2000; Terriou *et al.*, 2000), although conflicting reports have found no improvement in human sperm motility at the same concentrations (Mathieu *et al.*, 1994; Tournaye *et al.*, 1994) and higher concentrations of 10 mM have been used to examine its effects on spermatozoa DNA damage (Banihani *et al.*, 2018). Other such PDE inhibiting compounds included Milrinone, a PDE3 inhibitor shown to effect human spermatozoa motility at 50 μM (Lefièvre *et al.*, 2002), and rolipram, a PDE4 inhibitor with effects at 10 µM (Marechal *et al.*, 2017). Sildenafil and its analogue Vardenafil were also screened without appearing as a hit. PDE5 is expressed at low levels in human spermatozoa (Lefièvre *et al.*, 2002) and its inhibition, *in vitro*, using sildenafil can improve sperm motility. However, conflicting studies reported that this effect requires vastly different concentrations. Lefievre *et al.* (2000) report that an increase in progressive motility required concentrations of at least 100 μM, while Glenn *et al.* (2007) report an improvement in progressive motility with just 0.67 μM (Lefievre *et al.*, 2000; Glenn *et al.*, 2007).

A substantial advantage of phenotypic screening is that it potentially opens new areas for investigation to improve our understanding of cell (Johnston *et al*., 2022). In this screen, in addition to those addressed above, there are several examples that warrant further investigation. For instance, enhancement of sperm motility by Linsitinib (Supplementary Movie S1, Fig. 2D–E) that selectively inhibits IGF-1R and the insulin receptor, is in keeping with the recent data of insulin modulating human sperm survival (Aitken *et al.*, 2021). Another novel consistent finding was that modulation of γ-Aminobutyric acid-GABA resulted in an increase in sperm motility (Supplementary Table SI). While there is significant literature on the role of GABA in induction of the acrosome reaction there is little relating to human sperm motility. In the current data, GABAalpha2/alpha3 agonist and NS11394 (a GABA_A_ receptor modulator) significantly increased sperm motility. Both are selective positive allosteric modulators of GABA_A_ receptors, albeit working on different GABA_A_ receptor subtypes. Usually, they are inert in the absence of GABA or an equivalent agonist. Moreover, TP003 and U90042, also GABA_A_ receptor agonists, were identified. More detailed experiments examining modulation of GABA and associated receptor complexes will uncover as yet undetermined biology related to human sperm motility.

In summary, using a novel HTS, we identified a large number of compounds that increased sperm motility. In addition to furthering our understanding of human sperm function, for example identifying new avenues for discovery such as the role of GABA in sperm motility, we highlighted PDE10A inhibitors as promising starting point for a medicinal chemistry programme for potential enhancement of male fertility. Moreover, with full disclosure of the results of screening (Supplementary Table SI), we present a detailed resource to inform further work in the field.

## Supplementary data

Supplementary data are available at *Human Reproduction* online.

## Data availability

The data underlying this article are available in the article and in its online supplementary material.

## Supplementary Material

deac007_Supplementary_Movie_1

deac007_Supplementary_Table_1

deac007_Supplementary_Table_2
